# *Pasteurella* species bloodstream infections in Queensland, Australia, 2000–2019

**DOI:** 10.1007/s10096-022-04411-w

**Published:** 2022-02-01

**Authors:** Kevin B. Laupland, Adam G. Stewart, Felicity Edwards, Patrick Harris, Claire Heney, Narelle George, Sonali Coulter, David L. Paterson

**Affiliations:** 1 Department of Intensive Care Services, Level 3 Ned Hanlon Building, Royal Brisbane and Women’s Hospital, Butterfield Street, Brisbane, Queensland 4029 Australia; 2 Queensland University of Technology (QUT), Brisbane, Queensland Australia; 3 Faculty of Medicine, UQ Center for Clinical Research, University of Queensland, Brisbane, Australia; 4 Infectious Diseases Unit, Royal Brisbane and Women’s Hospital, Brisbane, Australia; 5 Department of Microbiology, Pathology Queensland, Brisbane, Australia; 6 Medication Services Queensland, Chief Medical Officer and Health Regulation Branch, Brisbane, Australia

**Keywords:** *Pasteurella*, Bloodstream infections, BSI, Incidence, Bacterial infection, Bacteremia

## Abstract

*Pasteurella* species are infrequent but potentially severe causes of bloodstream infection (BSI). The objective of this study was to determine the incidence, risk factors, and outcomes of *Pasteurella* species BSI in a large Australian population. Retrospective, laboratory-based surveillance was conducted in Queensland, Australia (population ≈ 5 million) during 2000–2019, and clinical and outcome information was established by linkage to state hospital admissions and vital statistics databases. During more than 86 million person-years of surveillance, 272 incident *Pasteurella* species BSI occurred for an overall age- and sex-standardized annual incidence of 3.3 per million residents. The incidence of *Pasteurella* species BSI was highest in recent years and older individuals were at greatest risk. The median (interquartile range) Charlson Comorbidity Index was 2 (0–4) with scores of zero, 1, 2, and 3 + observed in 81 (30%), 37 (14%), 44 (16%), and 110 (40%) of cases. The 30-day all-cause case fatality was 9% (24/272) and patients who died had more comorbidities and were less likely to have community-associated disease. Although *Pasteurella* species are infrequent causes of BSI, older individuals and those with comorbidities are at highest risk. The burden of this disease may be expected to increase with an aging and more comorbid population.

## Introduction

*Pasteurella* species, most commonly *P. multocida*, are infrequent but often serious causes of bloodstream infections (BSIs) [1]. These organisms colonize the upper aero-digestive tract of a range of wild and domestic animals, and human infection commonly arises as a result of wound infection following a bite or other direct exposure to oral secretions [1]. A case series and systematic review including 119 adult *Pasteurella* species BSI cases reported in the English language, peer-reviewed literature during 1951–2017 has been recently published [2]. These authors found that two-thirds of cases had comorbid medical illnesses, most notably liver disease, immune suppression, and malignancies, and that the BSIs most commonly were related to soft tissue and pleuropulmonary infections [2]. Furthermore, they reported a 30-day all-cause case-fatality rate of 31%, and that a major comorbid illness was the main risk factor for adverse outcome.

Knowledge surrounding the occurrence and determinants of an infectious disease is important to establish its burden of illness and define its natural history [3]. However, the body of literature investigating the epidemiology of *Pasteurella* species BSI is based on small case reports and series [2, 4–8]. The objective of this study was therefore to define the incidence, risk factors, and outcome of *Pasteurella* species BSI in a large population-based cohort in Australia.

## Methods

The study population included all residents (2019 population ≈ 5 million) of Queensland, Australia. Queensland has both public and private healthcare services with the publicly funded system adminstered through 16 hospital and healthcare service (HHS) regions [9]. All Queensland residents with incident BSI due to *Pasteurella* species identified within the publicly funded system between January 1, 2000, and December 31, 2019, were included. The human research ethics committee at Royal Brisbane and Women’s Hospital approved this study and granted a waiver of individual consent (LNR/2020/QRBW/62494).

All blood culture testing within the publicy funded healthcare system, including from community and institutional collection sites statewide, is performed by Pathology Queensland. Pathology Queensland used the BACT/ALERT® 3D system (bioMérieux, Durham, NC) throughout the study period with the exception that the BACT/ALERT® VIRTUO®system (bioMérieux, Durham, NC) that was implemented at the main central laboratory in 2018 that manages culture submissions from the Greater Brisbane area and several rural Queensland sites. Blood cultures were incubated for 5 days before being discarded if no growth was detected. BacT/ALERT FA plus (aerobic), FN plus (anaerobic), and PF plus (pediatric) media bottles were used for culture. Species identification methods included VITEK® GN ID, API 20E, and MALDI-TOF MS. Antibiotic susceptibility testing was performed using both an automated method (i.e., VITEK® AST card) and disc diffusion according to recognized standards (CLSI or EUCAST) at the time of testing.

All blood cultures with growth of *Pasteurella* species were retrospectively identified by the Clinical Information Systems Support Unit, Queensland Health. Incident BSIs were defined by the first isolation of a *Pasteurella* species per patient with all subsequent isolations of the same species within 30 days deemed to represent the same episode. Polymicrobial infections were those where a *Pasteurella* species was co-isolated with one or more other significant pathogens within a 48-h period [10].

Clinical and outcome information was obtained through linkages to statewide databases. A linkage was performed with the Queensland Hospital Admitted Patient Data Collection (QHAPDC) in order to obtain all healthcare encounters associated with both private and public institutions within the 2 years prior to, and 1 year following an index blood culture. Hospital admission and discharge dates, discharge survival status, and all diagnostic codes (ICD-10AM) were obtained. Multiple admission episodes occurring within a continuous time period (such as with inter-hospital transfers) were deemed to represent a single hospital admission for purposes of length of stay. The Registry of General Deaths was queried as of December 31, 2020, to confirm deaths in any setting within Queensland.

Bloodstream infections were classified as hospital-onset if the index blood culture was drawn two calendar days after admission or within two calendar days of hospital discharge [11]. Bloodstream infections diagnosed within the community or within the first two calendar days of stay in hospital were classified as community-onset. Healthcare-associated BSIs were those community-onset BSIs that occurred among nursing home residents, and those who had encounters at a healthcare institution within 30 days and/or admission to hospital for more than 2 days within the 90 days prior to index blood culture [11]. Community-onset BSIs that did not fulfill criteria for healthcare-associated infections were classified as community-associated. Comorbid medical illnesses were defined using the Charlson Comorbidity Index [12, 13]. A clinical focus was assigned based on review of diagnosis-related group and primary diagnosis hospital discharge codes.

Data was analyzed using Stata 16.1 (StataCorp, College Station, USA). The primary unit of analysis was incident BSI episodes and were reported as age- and sex-standardized (to 2019 Queensland population) annual rates per million population. Nonresidents of Queensland were excluded. Denominator data was stratified by age, sex, and hospital and health service area was obtained from Queensland Health using data available from the Australian Bureau of Statistics [14]. The total annual number of sets of blood cultures performed by Pathology Queensland was obtained [15]. Incidence rate ratios (IRR) with exact 95% confidence intervals (CI) were calculated for group comparison. *p*-values < 0.05 were deemed to represent statistical significance for all comparisons.

## Results

During 86 million person-years of surveillance, 272 incident *Pasteurella* species BSI occurred among 263 individuals for an age- and sex-standardized incidence of 3.3 per million residents per year. Five subjects had second, three had third, and one had a fourth episode(s) of incident *Pasteurella* species BSI. Most of the isolates were *P. multocida* (240; 88%), with 8 (3%) *P. canis*, 3 (1%) *P. pneumotropica*, and one each of *P. aerogenes*, *P. dagmatis*, and *P. haemolytica*, with the remaining 18 (7%) not further speciated. Among the 272 incident cases, only 8 (3%) were of hospital-onset, with the remaining 264 (97%) of community-onset cases further classified as healthcare- or community-associated in 46 (17%) and 218 (80%), respectively.

### Secular changes in incidence

**Fig. 1:**
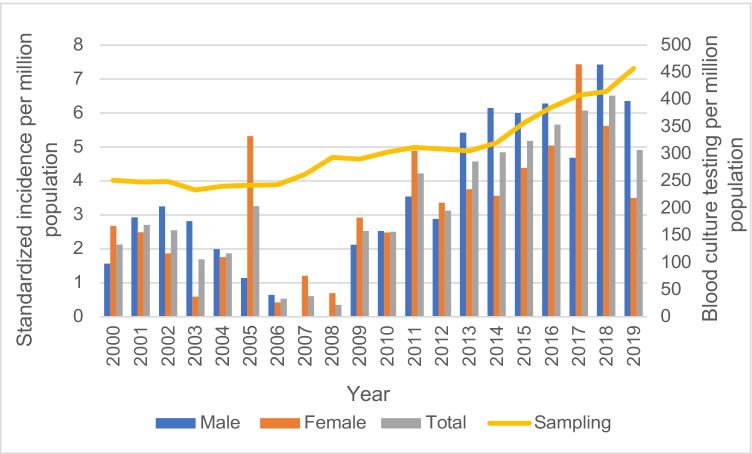
Incidence of *Pasteurella* species bloodstream infections in Queensland, 2000–2019

The incidence of *Pasteurella* species BSI varied during the 20 years of surveillance with the highest rates observed in the latter half of the study as shown in Fig. 1. Although the increase in standardized incidence observed in the latter study years paralleled higher sampling rates, in part related to few cases observed during 2006–2008, a linear relationship between incidence and sampling rate across the study years was not evident (Fig. 1).

### Demographic risk factors for acquisition

**Fig. 2:**
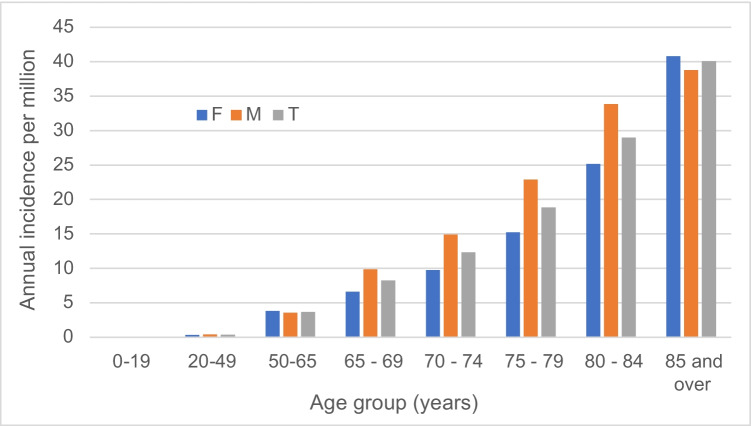
Age- and sex-specific incidence of *Pasteurella* species bloodstream infection in Queensland, 2000–2019

The median age was 75.0 (IQR, 64.7–83.4) years and 134 (49%) incident episodes were in females. There was a marked increase in incidence associated with advancing age as shown in Fig. 2. *Pasteurella* species BSI were rarely observed in younger individuals with incidence rates of 0.09 and 0.36 per million per year among those aged < 20 and 20–49 years of age, respectively. Overall, males and females were at similar risk for developing *Pasteurella* species BSI (3.3 vs. 3.1 per million, IRR for males 1.05; 95% CI, 0.82–1.34; *p* = 0.7). While the incidence of *Pasteurella* species BSI demonstrated moderate variation by hospital and health service area, there was no evident pattern of differential excess risk according to geographic region.

### Comorbid illnesses

Underlying medical illnesses were common among individuals with *Pasteurella* species BSI with median (IQR) Charlson Comorbidity Index scores of 2 (0–4). Scores of zero, 1, 2, and 3 + were observed in 81 (30%), 37 (14%), 44 (16%), and 110 (40%) of cases. The most common comorbidities identified were diabetes mellitus (93; 34%), congestive heart failure (56; 21%), and renal disease (52; 19%) as shown in Table 1.

**Table 1:** Clinicial features and outcome of *Pasteurella* species bloodstream infection in Queensland, 2000–2019

Factor	Survived (*n* = 248)	Died (*n* = 24)	Overall (*n* = 272)	*p*-value
Median age	74.8 (64.3–82.9)	79.2 (68.3–84.6)	75.0 (64.7–83.4)	0.3
Male sex	126 (51%)	12 (50%)	138 (51%)	0.6
Infection onset				0.012
Hospital	5 (2%)	3 (13%)	8 (3%)	
Community-associated	203 (82%)	15 (63%)	218 (80%)	
Healthcare-associated	40 (16%)	6 (25%)	46 (17%)	
Median Charlson	2 (0–4)	3 (2–5)	2 (0–4)	0.016
Charlson variables				
Myocardial infarction	16 (6%)	3 (13%)	19 (7%)	0.2
Congestive heart failure	49 (20%)	7 (29%)	56 (21%)	0.3
Peripheral vascular disease	13 (5%)	1 (4%)	14 (5%)	0.6
Cerebrovascular disease	12 (5%)	3 (13%)	15 (6%)	0.1
Dementia	11 (4%)	1 (4%)	12 (4%)	0.7
Chronic pulmonary	45 (18%)	5 (21%)	50 (18%)	0.5
Rheumatic	8 (3%)	0	9 (3%)	0.5
Peptic ulcer disease	7 (3%)	0	7 (3%)	0.5
Liver disease	24 (10%)	6 (25%)	30 (11%)	0.035
Diabetes mellitus	88 (35%)	5 (21%)	93 (34%)	0.2
Plegia	5 (2%)	3 (13%)	8 (3%)	0.025
Renal disease	44 (18%)	8 (33%)	52 (19%)	0.063
Malignancy	25 (10%)	5 (21%)	30 (11%)	0.11
HIV	0	0	0	
Focus of infection				0.2
No focus	113 (46%)	13 (54%)	126 (46%)	
Soft tissue	82 (33%)	2 (8%)	84 (31%)	
Bone and joint	5 (2%)	0	5 (2%)	
Head and neck	1 (< 1%)	0	1 (< 1%)	
Lower respiratory	21 (8%)	5 (21%)	26 (10%)	
Endovascular	1 (< 1%)	0	1 (< 1%)	
Central nervous system	1 (< 1%)	0	1 (< 1%)	
Abdominal	14 (6%)	3 (13%)	17 (6%)	
Urinary/pelvic	10 (4%)	1 (4%)	11 (4%)	

### Clinical determinants and outcome

All patients were admitted to hospital and had a median (IQR) length of stay of 8 (5–13) days. The most common focus of infection was soft tissue (84; 31%) followed by lower respiratory (26; 10%), and abdominal (17; 6%) as shown in Table 1. Polymicrobial infections occurred in 6 cases with 5 having one co-isolate and one two co-isolates. These organisms included one each of *Brevundimonas vesicularis*, *Proteus mirabilis*, *Shewanella algae*, *Staphylococcus aureus*, and *Streptococcus agalactiae*, and two unspeciated Gram-negative bacilli. Penicillin/ampicillin susceptibility testing results were available for 232 isolates for which resistance was found in only one case of pneumonia due to *P. aerogenes*. No resistance to ciprofloxacin was observed among 96 isolates tested.

Twenty-three (8%) patients died during their index hospital admission and the 30-day all-cause case fatality was 9% (24/272). Patients who died had more comorbidities and were less likely to have community-asociated disease as shown in Table 1. Although there was no overall association between case fatality and focus of infection, a higher proportion of deaths were observed in association with pulmonary or abdominal sources of infection (Table 1).

## Discussion

In this study, we report the incidence of *Pasteurella* species BSI and observe secular trends over a prolonged period in a large Australian population. We observed that there was an increasing incidence even after adjusting for changes in the population demography. This is of particular importance given that during 2000 to 2019, the Queensland population increased from 3.5 to 5 million residents with the subpopulation of the eldest residents tripling among males and more than doubling overall for both sexes. While the possibility exists that increased blood sampling could lead to an increase in detected cases, it is unlikely that this would explain the magnitude of the increase that we observed (Fig. 1). In any case, it is evident that the burden of confirmed disease due to *Pasteurella* species BSI is increasing in our population.

There is a paucity of previously published data to compare our incidence rates [16]. We previously estimated the incidence of *Pasteurella* species BSI as 1 case per million population in Calgary, Canada, using regional laboratory data during 2000 and 2001 [4]. Nseir et al. conducted a retrospective survey of all *P. multocida* infections in Israel during 2000–2005 and included 77 cases overall for an estimated incidence of 1.9 per million population annually [8]. Of this cohort, 25 were bacteremic corresponding to an incidence of BSI of 0.6 per million. It is noteable that their questionnaire response rate was 57% raising the possibility of missed cases and an underestimate of true incidence. Our study systematically identified cases during more than 80 million person-years of surveillance and as a result, we were able to assemble a sizable cohort of 272 cases. This study represents an important contribution to the literature as we triple the previously reported experience and lead further insight to epidemiology of these infections [2, 5].

It is important to note that we observed a much lower case-fatality rate as compared to the existing pool of literature. In the report conducted by Chatelier et al., the case-fatality rates in the case series and case report literature review were 15% and 34%, respectively [2]. While our lower rate could reflect population characteristics, improvements of treatment over time, or access to healthcare services, we suspect that the markedly higher case fatality observed in a summary of case reports relates to a bias whereby unusual or severe cases may be more likely to be both submitted and accepted for publication [17]. While this is potentially less of an issue for case series, single hospital-based studies are also subject to important selection biases [18]. Importantly, 2/25 (8%) of bacteremic cases died in the systematically sampled study reported from Israel by Nseir and colleagues which is very similar to our observation [8].

Like with previous reports, we found soft tissue and pleuropulmonary infections as the most common foci of *Pasteurella* species BSI [2]. In contrast, we rarely found endovascular infections or a central nervous system focus for *Pasteurella* species BSI. The presence of significant comorbidities among patients with *Pasteurella* species BSI is a commonality among our population and previous investigations by others. We observed that less than 1/3 of our cases were free of comorbid illness. The prevalence of diabetes, cancer, chronic lung disease, and kidney disease in the general Queensland population is approximately 4.5%, 3.4%, 1.6%, and 1%, respectively [19]. We observed much higher rates of these conditions in our cases suggesting that these factors may be associated with approximate 8-, 5-, 7-, and 19-fold higher risks for *Pasteurella* species BSI, respectively.

While our study has a number of strengths, there are some limitations that merit discussion. First, this study was retrospective and was limited to data already collected in existing databases. As a result, we were not able to tailor data collection to gather details on variables such as zoonotic exposures or antibiotic therapies [20]. More specifically, we are not able to examine the role of pet ownership or animal bites or how the use of post-bite antibiotic therapies may influence the incidence of these infections. Second, isolates were not available for further testing. Third, our study was limited to cultures performed within the publicly funded healthcare system and cases presenting to private hospitals were not included. While we suspect that this represents a relatively small proportion of cases overall, our incidence rates should be viewed as conservative estimates of the true number of cases occurring in Queensland. Fourth, like with all studies examining BSI, ascertainment of cases requires that a specimen of blood be submitted and subsequently cultured positive. There were no specific protocols directing physicians to order these tests and the possibility exists that bias could have been introduced in the study if decisions to order blood cultures varied among areas or over time (i.e., such as among the elderly with comorbid disease). Finally, as a result of the relatively small number of overall deaths (*n* = 24) that we observed, we had limited statistical power to examine determinants of outcome and multi-variable analysis was precluded.

In summary, this novel study details the epidemiology of *Pasteurella* species BSI in a large Australian population over two decades. *Pasteurella* species are important causes of BSI and the burden may be expected to increase in the coming years as the population ages and comorbid disease becomes more prevalent.

## Data Availability

Data cannot be shared publicly due to institutional ethics, privacy, and confidentiality regulations. Data release for the purposes of research under Sect. 280 of the Public Health Act 2005 requires application to the Director General (PHA@health.qld.gov.au).
